# Metformin relieves neuropathic pain after spinal nerve ligation via autophagy flux stimulation

**DOI:** 10.1111/jcmm.14033

**Published:** 2018-11-19

**Authors:** Weidong Weng, Chenglun Yao, Keshav Poonit, Xijie Zhou, Chao Sun, Feng Zhang, Hede Yan

**Affiliations:** ^1^ Department of Orthopedics Division of Plastic and Hand Surgery The Second Affiliated Hospital and Yuying Children’s Hospital of Wenzhou Medical University Wenzhou China; ^2^ Joseph M. Still Burn and Reconstructive Center Jackson Mississippi

**Keywords:** apoptosis, autophagy flux, metformin, neuropathic pain, spinal nerve ligation

## Abstract

Neuropathic pain is a well‐known type of chronic pain caused by damage to the nervous system. Autophagy is involved in the development and/or progression of many diseases, including neuropathic pain. Emerging evidence suggests that metformin relieves neuropathic pain in several neuropathic pain models; however, metformin's cellular and molecular mechanism for pain relief remains unknown. In this study, we investigated the therapeutic effects of metformin on pain relief after spinal nerve ligation (SNL) and its underlying mechanism of autophagy regulation. Behavioural analysis, histological assessment, expression of c‐Fos and molecular biological changes, as well as ultrastructural features, were investigated. Our findings showed that the number of autophagosomes and expression of autophagy markers, such as LC3 and beclin1, were increased, while the autophagy substrate protein p62, as well as the ubiquitinated proteins, were accumulated in the ipsilateral spinal cord. However, metformin enhanced the expression of autophagy markers, while it abrogated the abundance of p62 and ubiquitinated proteins. Blockage of autophagy flux by chloroquine partially abolished the apoptosis inhibition and analgesic effects of metformin on SNL. Taken together, these results illustrated that metformin relieved neuropathic pain through autophagy flux stimulation and provided a new direction for metformin drug development to treat neuropathic pain.

## 1 INTRODUCTION

Neuropathic pain is a common and refractory disease in clinical settings, which can be caused by injuries to the peripheral or central nervous system.^1^ Several systemic retrospective studies have demonstrated that patients with neuropathic pain have a lower quality of life than patients with chronic diseases, such as cancer, diabetes, chronic heart failure and stroke.^2^, ^3^ The pathophysiological mechanisms underlying neuropathic pain are not fully understood to date, which contributes to the challenge of managing patients.^4^, ^5^ Neuronal apoptosis in the spinal cord has been implicated in a cascade of events, which is involved in the generation and maintenance of neuropathic pain.^6^

Mitochondrial dysfunctions that lead to the activation of the apoptotic cascade are one of the key pathophysiological mechanisms associated with neuropathic diseases.^7^ Mitochondrial membrane permeabilization releases cytochrome C, which subsequently forms a complex that initiates the cleavage and activation of caspases.^8^, ^9^, ^10^ The spinal nerve ligation (SNL) peripheral neuropathy model activates a neuronal apoptotic cascade that includes the release of cytochrome C into the cytosol and caspase‐3 activation.^11^

Autophagy is a lysosomal degradation pathway and a homoeostatic cellular mechanism that is essential for survival, differentiation, development and homoeostasis.^12^ Autophagy is closely associated with apoptosis in the pathological process of many degenerative diseases, including osteoarthritis,^13^ age‐related macular degeneration^14^ and Alzheimer's disease.^15^ Meanwhile, recent investigations showed that autophagy was involved in neuropathic pain processing.^16^, ^17^, ^18^, ^19^, ^20^ As a result of the dysfunction of autophagy flux,^16^ increased markers of autophagy are observed in the early stages of SNL, which represents the accumulation of dysfunctional autophagosomes.^17^, ^18^ Furthermore, rapamycin, which is an autophagy inducer, induces long‐lasting analgesia, inhibits interleukin‐1β secretion,^19^ improves nerve myelination^20^ and prevents pain chronification. Accordingly, neuropathic pain was dramatically enhanced by autophagy inhibitors.^18^, ^20^

Metformin is one of the most widely used hypoglycaemic drugs for the treatment of type 2 diabetes. Metformin exhibits a variety of pharmacological properties, including anti‐inflammatory, anti‐cancer and antioxidant properties.^21^, ^22^ It was reported that metformin has been demonstrated to be a therapeutically effective drug candidate for various nervous system disorders, including Parkinson's disease,^23^ Huntington's disease^24^ and spinal cord injury.^25^ Similarly, metformin was reported to prevent etoposide‐induced neuronal apoptosis by inhibiting the mitochondrial permeability transition pore.^26^ Additionally, it has been demonstrated that metformin attenuates neuropathic pain through up‐regulation of AMPK after peripheral nerve injury.^27^ However, the molecular mechanism of metformin treatment in pain relief has not been completely defined. Specifically, the relationship between autophagy and apoptosis with the therapeutic effects of metformin in the neuropathic pain has not been previously investigated.

In this study, we examined metformin‐mediated effects on pain relief, apoptosis and autophagy activity in a model of SNL‐induced neuropathic pain using histological and protein analyses. Our data show that metformin may attenuate SNL‐induced mechanical and thermal hyperalgesia, as well as the expression of c‐Fos, a marker related to pain and other nociceptive stimuli. We also determined the signalling pathways and molecular mechanisms involved in neuronal apoptosis and autophagy following SNL, especially the pathways related to neuropathic pain. Collectively, our results suggest that metformin may be an effective and feasible target for drug development to treat neuropathic pain.

## 2 MATERIALS AND METHODS

### 2.1 Animals

Adult male Sprague‐Dawley rats weighing 220 to 250 g were purchased from the Animal Centre of the Chinese Academy of Sciences in Shanghai, China. All experimental procedures were approved by the Institutional Animal Care Committee of Wenzhou Medical University and performed according to the guidelines of the National Research Council for the care and use of laboratory animals. The animals were kept in specific, pathogen‐free conditions in 12‐hour day‐night cycles at 22‐24°C. All rats received food and water ad libitum.

### 2.2 Surgical procedure

Following the procedure originally proposed by Kim and Chung^28^ that was adapted to rats, the SNL model was used as a model of neuropathic pain. Briefly, rats were deeply anaesthetized with sodium pentobarbital (50 mg/kg body weight by intraperitoneal injection), and the left lumbar (L) 6 transverse process was carefully removed to visually identify the L4 and L5 spinal nerves. The L5 spinal nerve was isolated and tightly ligated with 4‐0 silk thread. Complete haemostasis was confirmed, and the wound was sutured. The procedure of the sham group was identical to the procedure for the experimental group, except the SNL was not performed.

### 2.3 Drug treatment

Metformin and chloroquine (CQ) were obtained from Sigma‐Aldrich (St. Louis, MO, USA) and dissolved in saline immediately prior to intraperitoneal (ip) administration, respectively. After surgery, the metformin solution was immediately injected intraperitoneally at a dose of 50 mg/kg/d until the rats were killed. A different group of rats was treated with 50 mg/kg/d metformin and 50 mg/kg/d CQ at the same time. Equivalent normal saline injections were administered for the vehicle controls. All animals showed no significant side effects, such as an increase in mortality or infectious diseases, resulting from drug treatment during these experiments. Drug dosages were selected based on data from previous studies and preliminary experiments.^25^

### 2.4 Behavioural tests

Before behavioural testing, animals were habituated to testing circumstance for 2 days. Mechanical withdrawal threshold (MWT) and thermal withdrawal latency (TWL) tests were performed on pre‐operative day 2 and post‐operative days 1, 3, 5, 7, 10, 14, 17 and 21. Each animal was placed in a Plexiglas chamber and habituated for 1 hour prior to each test session before and after drug treatment. The behavioural investigators were blinded to the drug administration conditions.

The MWT test was carried out to assess the response of the paw to a mechanical stimulus. Each rat was placed in a small Plexiglas cage with a metal mesh floor. The rigid tip of the 2390 series electronic Von Frey anaesthesia meter (IITC Life Science Inc, USA) was placed onto the plantar surface of the hind paw and pressed upward slowly until a withdrawal reflex was observed, and the force that elicited the withdrawal reflex was recorded.^29^

To quantitatively assess TWL, rats were placed on the glass surface of a thermal testing apparatus (Model 336, IITC/Life Science Instruments, Woodland Hills, CA). A beam of radiant heat was applied to the left hind paw, and the time from irradiation to paw withdrawal was recorded. Each test session included the delivery of three thermal stimuli at 5 minutes intervals, and the mean latency was used. A cut‐off time of 30 seconds was set to avoid tissue damage.^30^

### 2.5 Specimen preparation

After completion of the behavioural tests on post‐operative day 7, the rats were killed by administering an overdose of sodium pentobarbital solution in each group. The rats were transcardially perfused with 200 mL normal saline. The L5 spinal cord segments were rapidly removed. For histological analysis, L5 spinal cord segments were post‐fixed in 4% paraformaldehyde for 24 hours, dehydrated in 30% sucrose in PBS at 4°C for 24 hours and embedded in paraffin. Transverse sections (5‐μm thick) were mounted on slides for staining. For a Western blot test, the tissues were placed in liquid nitrogen immediately, and the ipsilateral L5 spinal cords were separated immediately and preserved at −80°C. Additionally, about a 1 × 1 mm segment was first harvested in the ipsilateral L5 segment of the spinal cord dorsal horn under microscopy visualization for transmission electron microscope study before Western blot analysis.

### 2.6 Immunofluorescence and immunohistochemistry

Transverse sections mounted on slides were prepared as described above. For immunofluorescence, transverse sections were treated with primary antibodies targeting NeuN (1:400, Abcam), LC3 (1:400, Sigma‐Aldrich), p62 (1:300, Cell Signaling Technologies) and cleaved caspase‐3 (1:300, Cell Signaling Technologies). The sections were washed four times with phosphate buffered saline containing 0.05% Tween‐20 (PBST) and incubated with AlexaFluor 568, AlexaFluor 488 or AlexaFluor 647 donkey anti‐rabbit/mouse secondary antibodies for 1 hour at 37°C. Afterward, the sections were washed with phosphate‐buffered saline (PBS), incubated with 4′,6‐diamidino‐2‐phenylindole (DAPI) for 7 minutes, rinsed with PBS and sealed with a coverslip. Images were captured using a confocal fluorescence microscope (Nikon, Japan). For immunohistochemistry, the transverse sections of the spinal cord were treated with primary antibodies targeting c‐Fos protein (1:200, Abcam) and were incubated with horseradish peroxidase‐conjugated secondary antibodies overnight at 4°C. Next, the sections were developed with 3,3′‐diaminobenzidine and counterstained with haematoxylin. Images were captured with the aid of a light microscope (Olympus, Japan).

### 2.7 Western blot analysis

For protein extraction, the specimens were homogenized in modified buffer (50 mmol/L Tris‐HCl, 1% NP‐40, 20 mmol/L DTT, 150 mmol/L NaCl, PH = 7.4) containing a protease inhibitor cocktail (10 μl/mL, GE Healthcare Biosciences, PA, USA). The BCA method was used to determine the total protein concentration. An equivalent of 60 μg of total protein were separated on a 12% (w/v) gel and transferred to PVDF membrane (Bio‐Rad). After blocking the membranes with 5% (w/v) non‐fat milk for 2 hours, they were incubated with the following antibodies: Bax, cleaved caspase 3, Bcl‐2, Beclin1, p62, ubiquitin (1:1000; Cell Signaling Technology), LC3 (1:000; Sigma‐Aldrich), c‐Fos (1:000, Abcam) and GAPDH (1:200; Santa Cruz Biotechnology). The membranes were washed with TBS three times and incubated with secondary antibodies for 2 hours at room temperature. Signals were visualized using the ChemiDoc^TM^ XRS + Imaging System (Bio‐Rad), and the band densities were quantified with Image Lab 3.0 software (Bio‐Rad).

### 2.8 Transmission electron microscopy study

After fixation in 2.5% (w/v) glutaraldehyde overnight, the specimens were post‐fixed in 2% (v/v) osmium tetroxide and blocked with 2% (v/v) uranyl acetate. Tissues were embedded in araldite after dehydration in a series of acetone washes. Semi‐thin sections were cut and toluidine blue staining was performed for observation of localization. Finally, ultra‐thin sections from at least three blocks per sample were cut and observed using a transmission electron microscopy (TEM).

### 2.9 Statistical analysis

Data were expressed as the mean ± standard error of the mean (SEM). All analyses were conducted using SPSS 20 statistical software. Statistical significance was determined using Student's *t* test when there were two experimental groups. When more than two groups were compared, statistical evaluation of the data was performed with one‐way analysis of variance (ANOVA) followed by Dunnett's post‐hoc test. *P* < 0.05 was considered to be significant for all statistical comparisons.

## 3 RESULTS

### 3.1 Metformin attenuates SNL‐induced mechanical and thermal hyperalgesia

**Figure 1:**
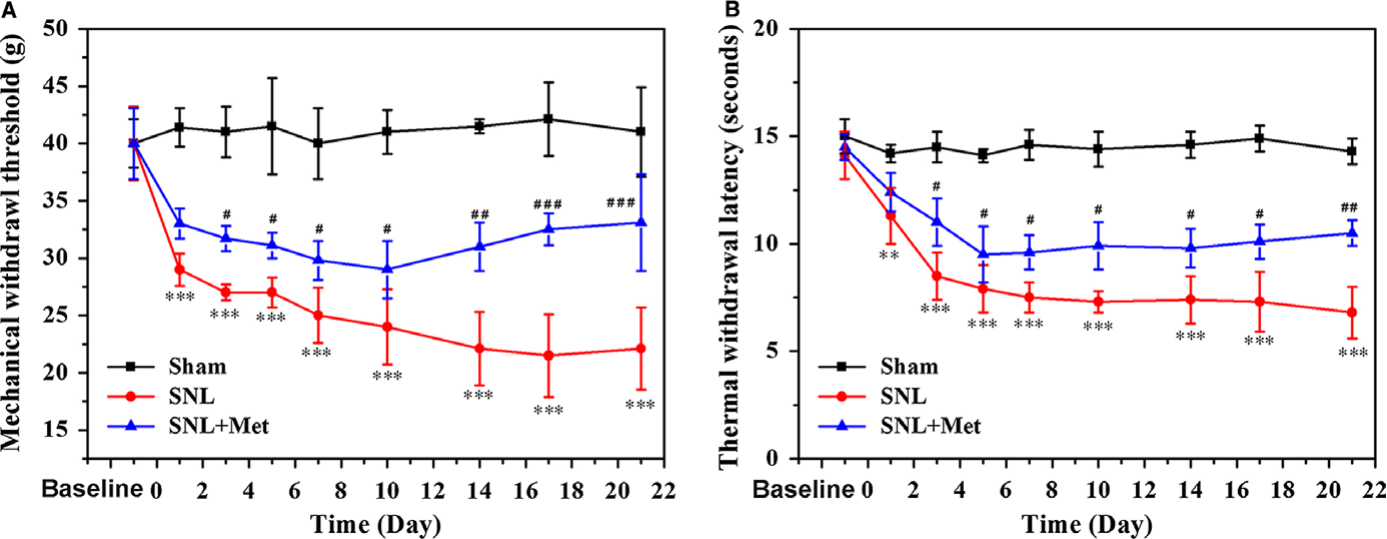
Metformin (Met, 50 mg/kg/d) attenuates SNL‐induced mechanical and thermal hyperalgesia. (A) The mechanical withdrawal threshold (MWT) tests and (B) thermal withdrawal latency (TWL) tests of rats in the sham group, SNL group and SNL + Met group. Values are expressed as the mean ± SEM, n = 5 per group. ^***^ *P* < 0.001, ^**^ *P* < 0.01, compared to sham group; ^###^ *P* < 0.001, ^##^ *P* < 0.01, ^#^ *P* < 0.05, compared to the SNL group

To evaluate the effects of metformin (50 mg/kg/d) on pain perception after SNL, MWT and TWL results were measured for 3 weeks. Behavioural tests were all conducted on the operated side. There were no significant differences in both baseline MWT and TWL among the different groups. The MWT and TWL did not change significantly from baseline during the testing period in the sham group. However, compared to the sham group, the MWT and TWL significantly decreased on post‐operative days 1‐21 in the SNL group (Figure 1A,B), which suggested that SNL‐induced persistent mechanical and thermal hyperalgesia in rats. When metformin administration started from the first post‐SNL day, no significant difference in MWT and TWL was exhibited between the SNL group and metformin‐treated group on day 1 post‐SNL. However, compared to the SNL group, the MWT and TWL were significantly higher on post‐operative days 3‐21 in the metformin‐treated group, which suggested that metformin treatment following SNL produced a persistent anti‐nociceptive effect.

### 3.2 Metformin decreases apoptosis in the spinal cord after SNL

**Figure 2:**
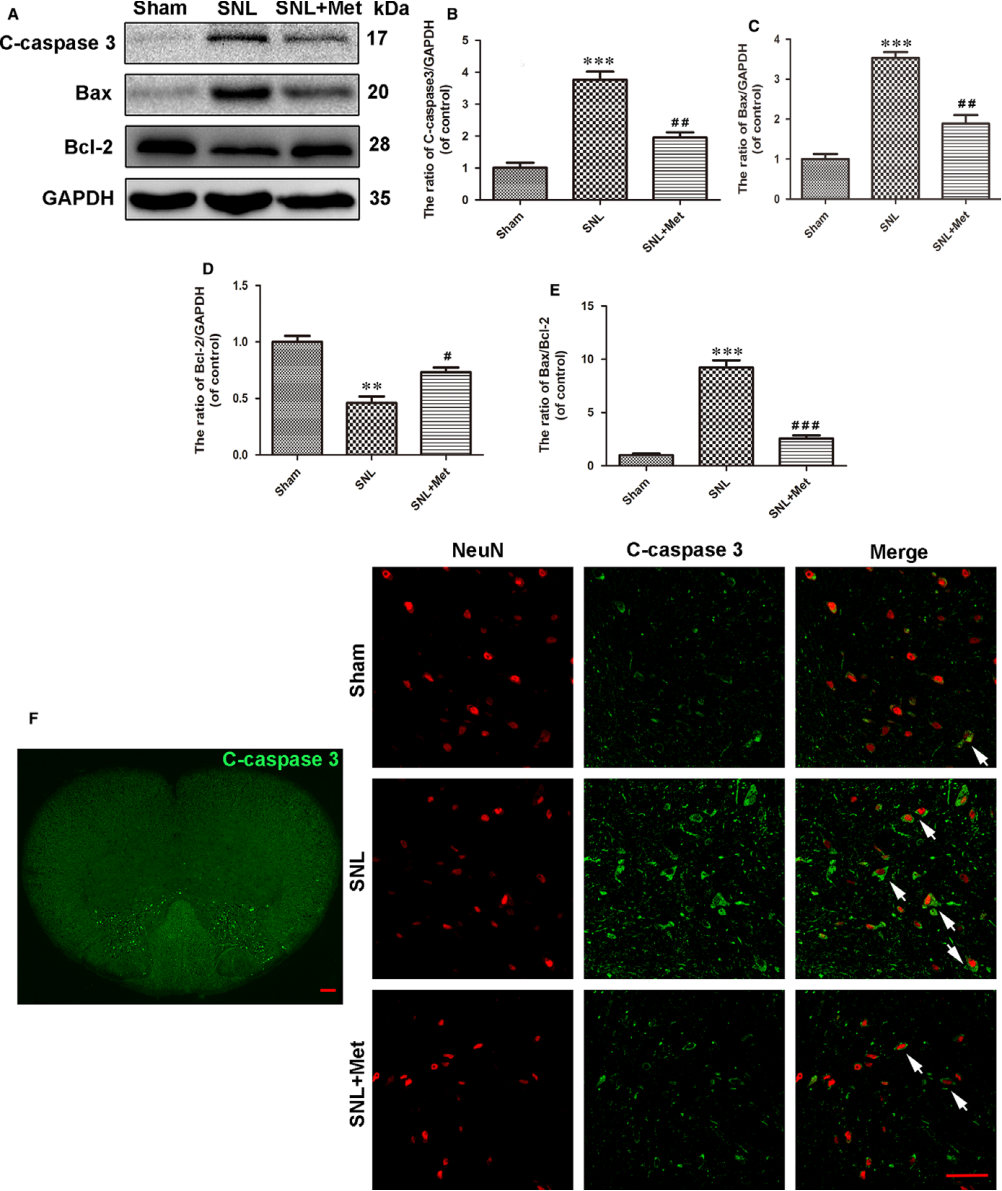
Metformin (50 mg/kg/d) decreases neuron apoptosis in the spinal cord after SNL. (A) Protein expression of C‐caspase 3, Bcl‐2 and Bax in rats from the sham, SNL and SNL + Met groups, respectively. GAPDH was used as the control and for band density normalization. (B‐E) The optical density analysis of C‐caspase 3, Bax and Bcl‐2 proteins, along with the Bax/Bcl‐2 ratio. (F) Distribution of C‐caspase 3 (green) immunoreactivity in the L5 spinal cord following SNL. Scale bar = 100 μm. Immunofluorescence images show that C‐caspase 3 (green) colocalize in neuron (NeuN, red) in the dorsal horn of L5 spinal cord (The white arrows indicate their co‐localization). Scale bar = 50 μm. Values are expressed as the mean ± SEM, n = 5 per group. ^***^ *P* < 0.001, ^**^ *P* < 0.01, compared to the sham group; ^###^ *P* < 0.001, ^##^ *P* < 0.01, ^#^ *P* < 0.05, compared to the SNL group

To evaluate the role of metformin (50 mg/kg/d) in modulating apoptosis after SNL, the expression levels of apoptosis‐related proteins (cleaved caspase 3, Bax and Bcl‐2) and double staining for NeuN (red) and cleaved caspase‐3 (green)were performed. Western blot analysis of cleaved caspase 3 showed that metformin significantly reversed the increasing levels of cleaved caspase‐3 caused by SNL (Figure 2A,B). Similarly, metformin treatment markedly down‐regulated the ratio of Bax/Bcl‐2 and the level of the proapoptotic protein Bax and up‐regulated the level of anti‐apoptotic protein Bcl‐2 (Figure 2A,C‐E). As is shown in Figure 2F, cleaved caspase‐3 was mainly expressed in the superficial laminae of L5 dorsal horn following SNL; double staining for NeuN and cleaved caspase‐3 was performed to observe the apoptosis level in the dorsal horn of the L5 spinal cord. In contrast, metformin treatment prevented SNL‐induced apoptosis. Taken together, these data suggest metformin administration is an effective strategy for inhibition of apoptosis after SNL.

### 3.3 Metformin promotes autophagy flux in the spinal cord after SNL

**Figure 3:**
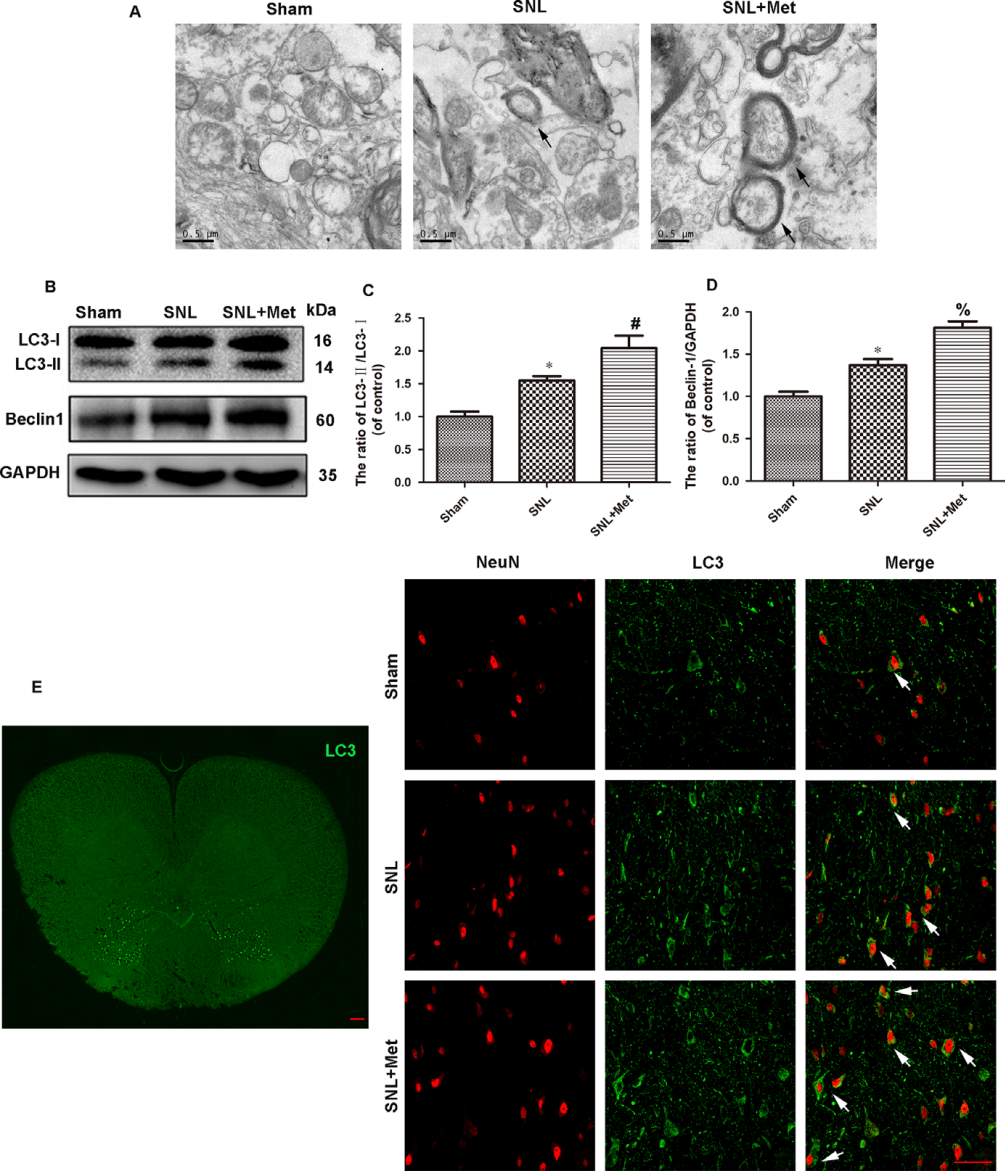
Metformin (50 mg/kg/d) promotes autophagy in the spinal cord after SNL. (A) Transmission electron microscopy showed the autophagosomes (black arrow), and the scale bars indicate 0.5 μm. (B) Protein expressions of LC3II/I and Beclin1of rats in sham, SNL and SNL + Met groups, respectively. GAPDH was used as the control and band density normalization. (C, D) The optical density analysis of LC3II/I and Beclin1 proteins. (E) Distribution of LC3 (green) immunoreactivity in the L5 spinal cord following SNL. Scale bar = 100 μm. Immunofluorescence images show that LC3 (green) colocalizes in neuron (NeuN, red) in the dorsal horn of the L5 spinal cord (The white arrows indicate their co‐localization). Scale bar = 50 μm. Values were expressed as the mean ± SEM, n = 5 per group. ^*^ *P* < 0.05, compared to the sham group; ^#^ *P* < 0.05, compared to SNL group; ^%^ *P* > 0.05, compared to SNL group

**Figure 4:**
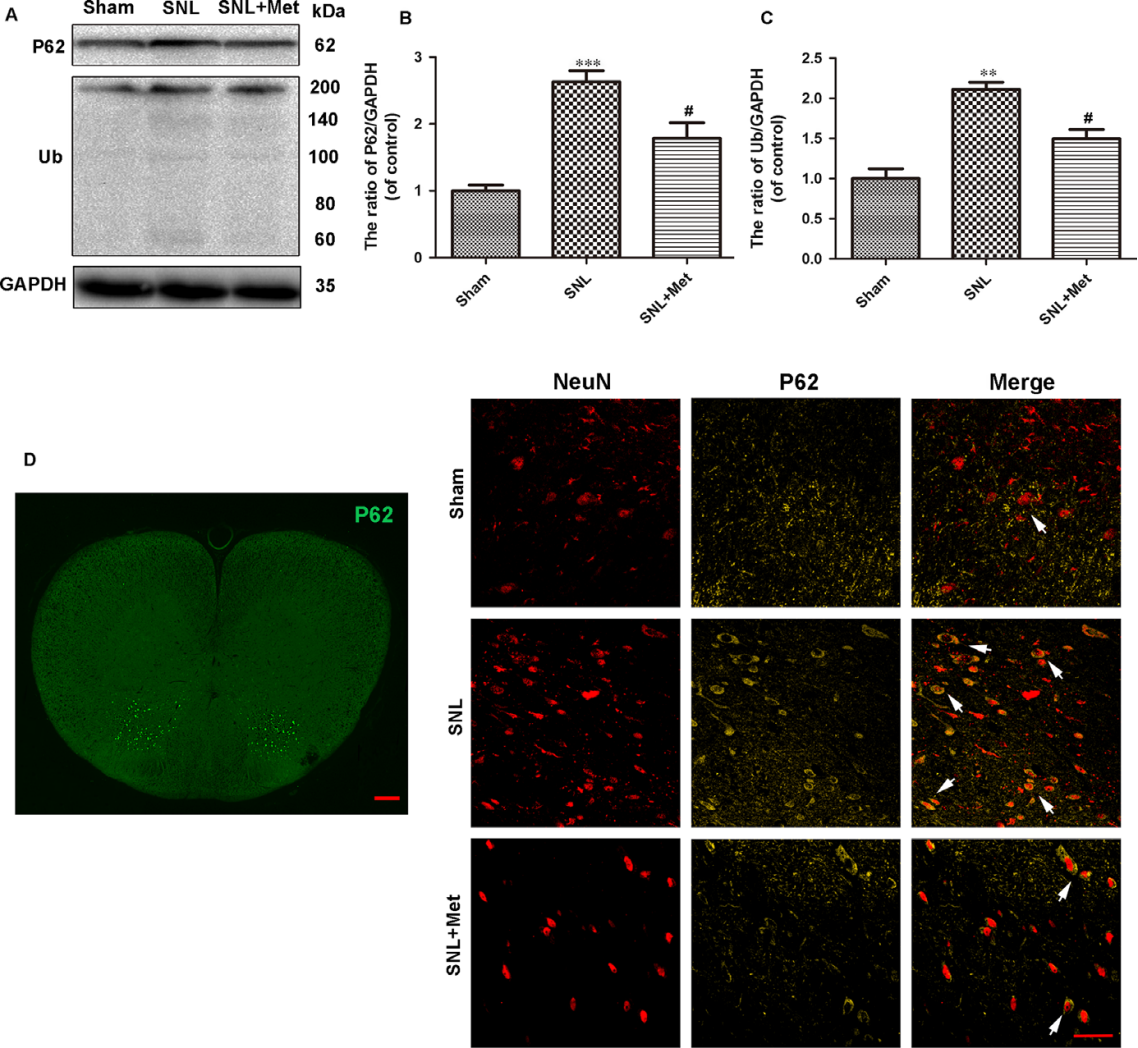
Metformin (50 mg/kg/d) promotes autophagy flux in the spinal cord after SNL. (A) Protein expression of P62 and ubiquitinated proteins (Ub) of rats in the sham, SNL and SNL + Met groups, respectively. GAPDH was used as the control and for band density normalization. (B, C) The optical density analysis of P62 and ubiquitinated proteins. (D) Distribution of P62 (green) immunoreactivity in the L5 spinal cord following SNL. Scale bar = 100 μm. Double staining for NeuN (red)/P62 (yellow) of sections from the dorsal horn of the L5 spinal cord (The white arrows indicate their co‐localization). Scale bar = 50 μm. Values are expressed as the mean ± SEM, n = 5 per group. ^***^ *P* < 0.001, ^**^ *P* < 0.01, compared to the sham group; ^#^ *P* < 0.05, compared to the SNL group

To determine the morphological signatures of autophagy in neurons, autophagosomes were observed by TEM, which is a standard method to check autophagy activation. As shown in Figure 3A, the SNL and metformin‐treated groups had many autophagosomes compared to the sham group. To molecularly confirm the induction of autophagy, we examined the expression of autophagy by Western blot and double staining for NeuN (red)/LC3 (green). Autophagosome formation is mediated by conjugation systems composed of ATG proteins, which culminate in the conjugation of ATG12 to ATG5 and conversion of a soluble form of microtubule‐associated protein 1 light chain 3 (LC3‐I) to another bound form, LC3‐II. Thus, the ratio of LC3II/LC3I is a marker of autophagy in some studies.^31^, ^32^ Compared to the SNL group, the ratio of LC3II/LC3I was lower in the sham group but was significantly higher in the metformin treatment group (Figure 3B,C). As is shown in Figure 3E, LC3 was mainly expressed in the superficial layer of L5 dorsal horn following SNL; double staining for NeuN (red)/LC3 (green) was performed to observe the autophagy in the dorsal horn of L5 spinal cord of each group. LC3 was accumulated in neurons in the SNL group, and there were no significant differences between the SNL group and the metformin‐treated group (Figure 3E). We also examined the expression of the protein Beclin‐1, which is a key protein in autophagy. A slight increase in Beclin‐1 was observed in SNL and metformin‐treated groups compared to the sham group. However, there were no significant differences in the level of Beclin‐1 between the SNL group and the metformin‐treated group (Figure 3B,D). However, increases in the level of autophagosomes or LC3II/LC3I can reflect either the induction of autophagy or inability to clean the autophagosome or amphisome.^33^ The adapter protein p62 (SQSTM1) mediates delivery of ubiquitinated proteins to autophagosomes, and the ubiquitinated proteins are degraded by autophagy. Because the protein p62 is a substrate of the autophagic process and its expression is considered a marker of autophagic flux, which should be accumulated when autophagy is impaired.^34^ As the results show, the SNL group had a higher level of p62 and ubiquitinated proteins than the sham group, which indicated that the initial accumulation of LC3 and autophagosomes after SNL reflected a decrease in autophagy flux. However, metformin treatment (50 mg/kg/d) reversed this effect (Figure 4A‐C). Immunofluorescent staining showed that p62 was mainly expressed in superficial layer of L5 dorsal horn following SNL. To observe the autophagy flux in neurons, double staining for NeuN (red)/p62 (yellow) in the dorsal horn of the L5 spinal cord was performed, which verified the same results (Figure 4D). These findings showed that the autophagy flux was disrupted following SNL, while metformin treatment corrected this disruption.

### 3.4 Metformin reduces the expression of c‐Fos in the spinal cord after SNL

**Figure 5:**
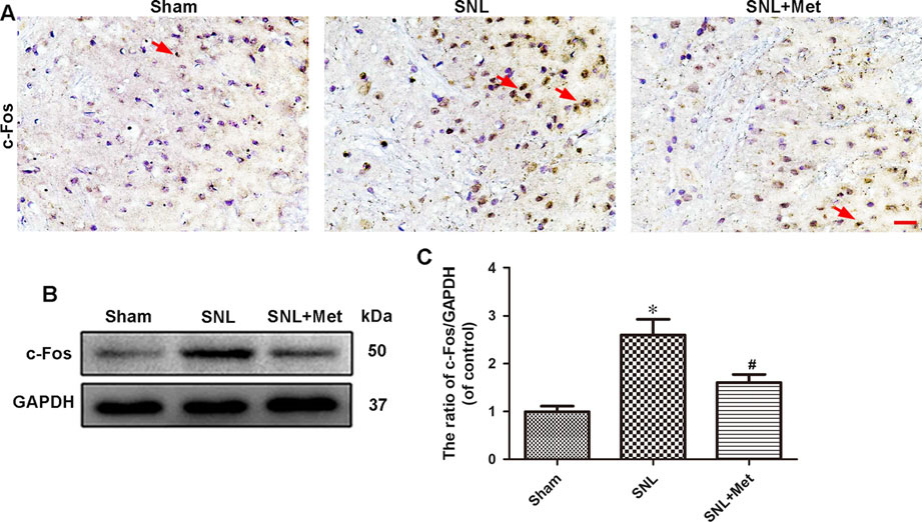
Metformin (50 mg/kg/d) reduces the expression of c‐Fos in the spinal cord after SNL. (A) Immunohistochemistry staining of c‐Fos in the dorsal horn of the L5 spinal cord. The red arrows show the neuron cells with positive staining for c‐Fos. Scale bar = 20 μm. (B) Protein expression of c‐Fos of rats from the sham, SNL and SNL + Met groups, respectively. GAPDH was used as the loading control and for band density normalization. (C) The optical density analysis of the c‐Fos protein. Values are expressed as the mean ± SEM, n = 5 per group. ^*^ *P *< 0.05 vs the sham group; ^#^ *P *< 0.05 vs the SNL group

To further evaluate the pain state at the molecular level, we examined the expression of c‐Fos in the dorsal horn of the L5 spinal cord, which was reported as a pain‐related protein.^35^, ^36^, ^37^, ^38^ The results of immunohistological analysis of c‐Fos showed only very few neuron cells which had positive c‐Fos staining in the sham group. Metformin treatment (50 mg/kg/d) significantly decreased the number of c‐Fos positive cells in the spinal cord following SNL (Figure 5A). These findings correlated with the results of the Western blot assay, which showed the expression level of c‐Fos in the L5 spinal cord was lowest in the sham group and highest in the SNL group (Figure 5B,C). These results indicated that metformin inhibits the expression of c‐Fos in the spinal cord following SNL.

### 3.5 Anti‐apoptosis effect of metformin is related to the autophagy flux after SNL

**Figure 6:**
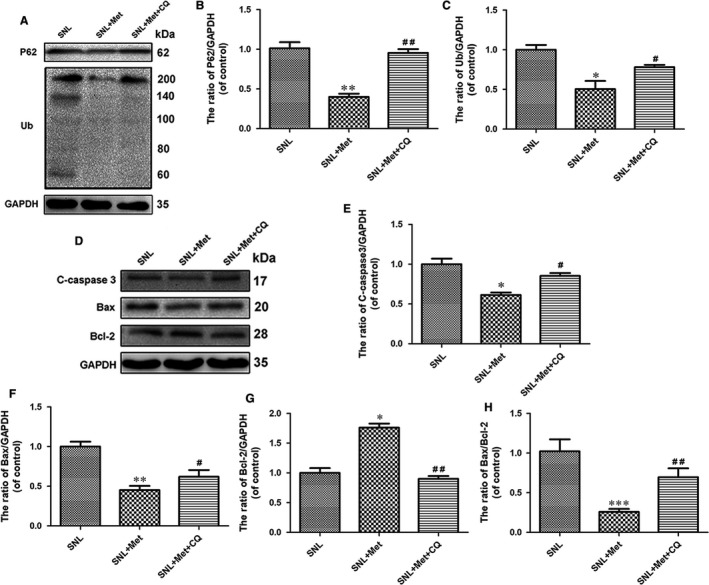
Anti‐apoptosis effect of metformin (50 mg/kg/d) is related to the autophagy flux after SNL. (A) The expression of p62 and ubiquitinated proteins in SNL, SNL + Met and SNL + Met + 3‐MA groups, respectively. (B, C) The optical density analysis of p62 and ubiquitinated protein proteins. (D) Protein expressions of C‐caspase 3, Bcl‐2 and Bax in the three groups. (E‐H) The optical density analysis of C‐caspase 3, Bcl‐2 and Bax proteins, along with the Bax/Bcl‐2 ratio. Values are expressed as the mean ± SEM, n = 5 per group. ^***^ *P* < 0.001, ^**^ *P* < 0.01, ^*^ *P *< 0.05 vs the SNL group; ^##^ *P* < 0.01, ^#^ *P *< 0.05 vs the SNL +Met group

To investigate whether autophagy flux was involved in the metformin‐induced anti‐nociceptive effect against apoptosis, CQ, which is a classic autophagy‐lysosome pathway inhibitor and is generally used to block autophagy flux,^39^, ^40^ was added to the metformin solution. A Western blot analysis was used to detect the expression of p62 and ubiquitinated proteins. As the results show, the expression of p62 and ubiquitinated proteins increased significantly after a combination of metformin (50 mg/kg/d) and CQ was used compared to metformin alone (Figure 6A‐C). Next, the degree of apoptosis was investigated using Western blotting to detect the levels of cleaved caspase 3, Bax and Bcl‐2 in the spinal cord. As the results show, we obtained the same results as reported for the metformin treatment group compared to the SNL group. Meanwhile, a combination of metformin and CQ evidently up‐regulated the levels of cleaved caspase 3, Bax and the ratio of Bax/Bcl‐2 and down‐regulated the level of Bcl‐2 compared to the metformin treatment group (Figure 6D‐H). Our results showed that the anti‐apoptosis effect of metformin was related to the correction of dysfunction in the autophagy‐lysosome pathway.

### 3.6 Blockage of autophagy by CQ abolishes the analgesic effects of metformin

**Figure 7:**
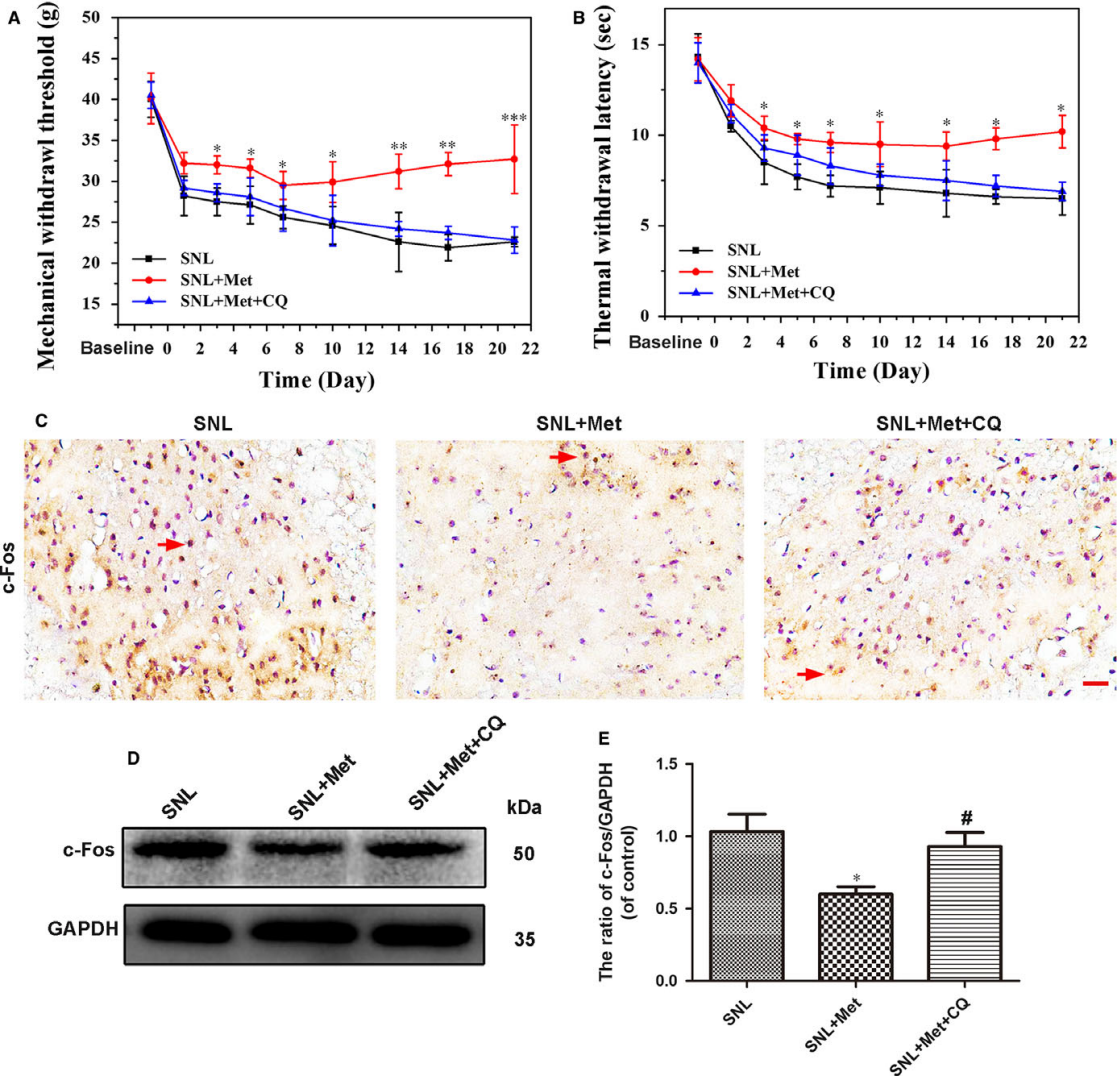
Blockage of autophagy flux by CQ abolishes the analgesic effect of metformin (50 mg/kg/d). (A) The mechanical withdrawal threshold (MWT) tests and (B) thermal withdrawal latency (TWL) tests of rats in the SNL group, SNL + Met group and SNL + Met + 3‐MA group. (C) Immunohistochemistry staining of c‐Fos in the three groups. The red arrows show the neuron cells with positive staining for c‐Fos. Scale bar = 20 μm. (D) Protein expression of c‐Fos in the SNL, SNL + Met and SNL + Met + 3‐MA groups, respectively. (E) The optical density analysis of c‐Fos protein. Values are expressed as the mean ± SEM, n = 5 per group. ^***^ *P* < 0.001, ^**^ *P* < 0.01, ^*^ *P* < 0.05 vs the SNL group; ^#^ *P* < 0.05 vs the SNL + Met group

To determine whether the analgesic effects of metformin on pain perception were related to autophagy, behavioural tests and experiments testing the expression of the c‐Fos, a marker related to pain and other nociceptive stimuli, were performed. Mechanical withdrawal threshold and TWL tests revealed that the effect of metformin therapy (50 mg/kg/d) on pain perception was significantly suppressed by CQ (Figure 7A,B). The results of immunohistological analysis of c‐Fos showed a combination of metformin and CQ distinctly increased the number of c‐Fos positive cells in the dorsal horn of the L5 spinal cord (Figure 7C). The Western blot results also showed that combination therapy by CQ could increase the expression of c‐Fos, which was decreased by metformin treatment following SNL (Figure 7D,E).

## 4 DISCUSSION

Neuropathic pain, which is characterized by hyperalgesia, allodynia and spontaneous pain, often occurs as a result of injuries to the peripheral or central nerve system.^41^ After peripheral nerve injury, a substantial number of cellular and molecular changes occur in the peripheral nerve, DRG, spinal dorsal horn and supraspinal regions, which contribute to peripheral and central sensitivity.^42^, ^43^ Several studies have shown that metformin relieves neuropathic pain in several neuropathic pain models, including peripheral nerve injury^27^ and diabetes.^44^, ^45^ However, as a multifunctional factor, the protective effects of metformin on the autophagy‐induced inhibition of apoptosis observed in neuropathic pain after SNL is not fully understood to date. Our results demonstrated that metformin effectively restored the disrupted lysosomal function to attenuate apoptosis and eventually alleviated the mechanical allodynia and thermal hyperalgesia associated with SNL.

In this study, it was shown that by one day after SNL, mechanical allodynia and thermal hyperalgesia developed in the ipsilateral paw and increased until the last day of our experiment. The third week after intraperitoneal administration of metformin at a dose of 50 mg/kg beginning at the time of nerve injury (day 3) significantly attenuated mechanical allodynia and thermal hyperalgesia in SNL animals. Aside from quantitative assessment of mechanical allodynia or thermal hyperalgesia, c‐Fos, a marker related to pain and other nociceptive stimuli to nervous system, was selected to evaluate the pain status of SNL rats. Our findings in this study showed that metformin treatment significantly reduced SNL‐induced c‐Fos levels and c‐Fos staining in the dorsal horn of the L5 spinal cord. These findings imply that metformin may prevent neuropathic pain caused by SNL.

Apoptosis is known to be involved in neuropathic pain following spinal nerve injury.^6^ However, the relationship between neuronal apoptosis in the spinal cord and development of allodynia or hyperalgesia is not fully known yet. Apoptosis might trigger structural changes in neurons, increase the sensitivity of the nociceptive system and therefore increase the induction of hyperalgesia or allodynia.^46^ Apoptosis could be mediated through two pathways: An extrinsic pathway through a death receptor and an intrinsic pathway managed by mitochondria.^47^ Mitochondrial dysfunction caused by oxidative stress, including the decrease of Bcl‐2, as well as the release of Bax, triggers activation of the caspase family, which leads to apoptosis.^48^, ^49^ In this study, we found that both the level of cleaved caspase 3 and the ratio of Bax/Bcl‐2 were up‐regulated dramatically in the SNL group, and metformin administration could significantly decrease caspase 3 activation and attenuated the ratio of Bax/Bcl‐2. Furthermore, double staining (for NeuN/cleaved caspase‐3) also revealed that metformin treatment decreased the level of apoptosis in the spinal cord following SNL. Based on these data, we conclude that attenuating mitochondrial dysfunction and regulation of apoptotic pathways may have a role in the anti‐nociceptive effects of metformin in SNL rats.

Autophagy is a process of self‐digestion in which the cell degrades useless proteins and organelles to sustain cellular function.^50^ Increasing evidence has indicated that autophagy is correlated with neuropathic pain after peripheral nerve injury.^51^ Additionally, increased markers of autophagy after SNL^17^, ^52^ represent the accumulation of autophagosomes; however, the exact mechanism remains controversial. Whether the accumulation of autophagosomes is due to the enhanced autophagy flux and autophagosome synthesis or decreased flux is uncertain.

Autophagy flux is defined as progress of autophagy from the formation of autophagosomes to cargo delivery and degradation in the lysosomes by lysosomal proteases.^53^, ^54^ Increased expression of autophagic hallmarks is not sufficient to conclude whether there was activation of autophagy flux.^55^ Increases in the level of autophagosomes or LC3 can reflect either the induction of autophagy or block the autophagosome degradation pathway.^33^ Previous reports demonstrated that autophagic marker accumulation after SNL did not occur due to increased initiation of autophagy but rather occurred due to inhibition of autophagy flux and lysosomal dysfunction.^16^

In this study, our data demonstrated that autophagy markers are at least partially induced by suppression of the degradation pathway. LC3, which is a central protein in the autophagy pathway, functions in autophagy substrate selection and autophagosome biogenesis,^56^ whereas p62 is an endogenous autophagy substrate and polyubiquinated proteins are degraded by lysosomes.^57^ Our data show that LC3 accumulation was accompanied by significant elevation of p62 and polyubiquitinated protein levels in the SNL model, which indicates the degradation pathway was suppressed. Furthermore, metformin enhanced the protein abundance of LC3 while decreasing the level of p62 and ubiquitinated proteins compared to the SNL group, which suggested metformin may repair lysosome function and contribute to restoring the autophagy‐lysosome pathway; however, the exact mechanism remains unclear, and it needs to be clarified further.

An increasing number of studies report that there is a close biochemical crosstalk mechanism between apoptosis and autophagy^33^; however, few studies have examined this mechanism for neuropathic pain. To reveal the relationship between autophagy and apoptosis, CQ, which is a classic autophagy‐lysosome pathway inhibitor,^39^, ^40^ was used in our study. Our study showed that the analgesic effect was diminished when activation of the autophagy‐lysosomal pathway was inhibited, which indicated that the analgesic effect of metformin occurred through apoptosis inhibition. We suppose that one possible reason for this anti‐apoptosis effect of metformin is that metformin‐induced autophagy flux inhibits the level of apoptosis in the spinal cord after SNL. The chemical inhibitor, CQ, is a classic autophagy‐lysosomal pathway inhibitor, but it may also activate potential non‐specific effects. To accurately understand the function of autophagy after SNL, transgenic animals with autophagic defects, such as Atg5−/− mice, should be used in future studies.

In conclusion, this study detected the effects of metformin on pain relief, apoptosis and autophagy flux after SNL. Our results demonstrate that metformin may prevent dysfunction of the autophagy‐lysosome pathway after SNL and may provide potential therapeutic interventions for SNL.

## CONFLICT OF INTEREST

The authors declare no conflict of interest.
